# Towards Real-Time Detection of Freezing of Gait Using Wavelet Transform on Wireless Accelerometer Data

**DOI:** 10.3390/s16040475

**Published:** 2016-04-02

**Authors:** Saba Rezvanian, Thurmon E. Lockhart

**Affiliations:** School of Biological and Health Systems Engineering, Ira A. Fulton Schools of Engineering, Arizona State University, Tempe AZ 85287, USA; Saba.Rezvanian@asu.edu

**Keywords:** Parkinson’s disease, freezing of gait, continuous wavelet transform, wireless sensors, fall

## Abstract

Injuries associated with fall incidences continue to pose a significant burden to persons with Parkinson’s disease (PD) both in terms of human suffering and economic loss. Freezing of gait (FOG), which is one of the symptoms of PD, is a common cause of falls in this population. Although a significant amount of work has been performed to characterize/detect FOG using both qualitative and quantitative methods, there remains paucity of data regarding real-time detection of FOG, such as the requirements for minimum sensor nodes, sensor placement locations, and appropriate sampling period and update time. Here, the continuous wavelet transform (CWT) is employed to define an index for correctly identifying FOG. Since the CWT method uses both time and frequency components of a waveform in comparison to other methods utilizing only the frequency component, we hypothesized that using this method could lead to a significant improvement in the accuracy of FOG detection. We tested the proposed index on the data of 10 PD patients who experience FOG. Two hundred and thirty seven (237) FOG events were identified by the physiotherapists. The results show that the index could discriminate FOG in the anterior–posterior axis better than other two axes, and is robust to the update time variability. These results suggest that real time detection of FOG may be realized by using CWT of a single shank sensor with window size of 2 s and update time of 1 s (82.1% and 77.1% for the sensitivity and specificity, respectively). Although implicated, future studies should examine the utility of this method in real-time detection of FOG.

## 1. Introduction

Injuries associated with fall incidences continue to pose a significant burden to persons with Parkinson’s disease (PD) both in terms of human suffering and economic loss. Annual fall incidence rates range from 50% to 70% in patients with PD, and recurrent falls are a major cause of disability in PD [1]. The resulting loss of independence and treatment costs add substantially to the healthcare expenditures in PD which is estimated to be $27 billion annually in the U.S. [2]. This number may rise substantially in the coming decades as the entire U.S. population ages. Furthermore, recurrent falls usually occur later in PD [1,3]. Indeed, among the top three priorities presented to the National Institute of Neurological Disorders and Stroke (NINDS) Council [4] as final recommendations of critical needs for advancing PD research, is to develop effective treatments for dopa-resistant features of PD. These features include motor symptoms such as gait and balance problems and freezing of gait leading to falls.

Freezing of gait (FOG) is one of the cardinal symptoms of the PD which is defined as an inability of a person to move one’s feet in spite of the fact that he/she intends to move [5,6]. FOG usually occurs during gait initiation and turning or, encountering an obstacle [7]. The occurrence of FOG increases at the later stage of PD and, the PD patients with mild and advanced stages experience FOG about 10% and 80%, respectively [8]. Not only FOG causes a negative impact on the activity of daily living, but also it is a common cause of falls in this population [9]. As such, specifying a treatment and finding a biomarker of FOG is considered as a goal of the PD clinical research.

Several questionnaires have been utilized to assess the severity of FOG conditions. One of the well-known and widely used questionnaires is Unified Parkinson’s Disease Rating Scale (UPDRS), Activities of Daily Living (ADL) part 14 [10]. This questionnaire rates FOG from scale 0 (no FOG) to 4 (frequent falls from freezing) based on the patient history. The accuracy and validity of this method for detecting FOG is completely subjective and dependent on patient and caregiver’s assessments and, they are not as accurate as the objective methods [11]. As such, objective measures of FOG have been developed and employed utilizing wearable sensors. These methods use pressure sensors or inertial monitoring units (IMU) along with waveform analysis to characterize the episodes of FOG. These studies indicate that the spectral component in the range of 3–6 Hz was associated with FOG episodes [12]. Recent wearable sensors, such as inertial measurement unit, could quickly and inexpensively deliver accurate measurements. Due to the form factor they also enable users to wear sensors on the various parts of body [13]. Additionally, these sensors can be used in any environment rather than the controlled environments [14]. The portability and widespread use of cell phones (with embedded IMUs) may make this method useful and universal.

Furthermore, sensor placement locations as well as calibration methods may influence the accuracy of FOG detection [7]. As such, there are several requirements that must be considered prior to designing any real-time FOG detection systems—such as minimum sensor nodes, sensor placement locations, and appropriate sampling period and update time [15,16]. For example, FOG episodes have different durations (from 0.5 s to 128 s) [15,16], and given a short duration of the FOG episodes, use of fast Fourier transform (FFT) with the minimum sample window size of 4 s will erroneously miss the FOG in the signal. However, methods such as continuous wavelet transform (CWT) which employ time domain information in a smaller sample window size may detect the short-duration FOG better than FFT method.

The objective of this study is to assess the effects of sampling duration and update time using the CWT for correctly identifying the FOG events in lieu of different sensor placements. The advantage of this method in comparison to other waveform methods (e.g., FFT) is that the CWT method uses both time and frequency components of a waveform and may improve the accuracy of FOG detection. Unlike Fourier transform, the continuous wavelet transform could construct a time-frequency representation of a signal which delivers the time and frequency localization. Furthermore, continuous wavelet transform (CWT) could assess how the frequency content (the power amplitude of specific frequency) of a signal changes over time. This detailed time-frequency analysis provides the ability to localize the transient state of a signal in time well better than FFT method. It is hypothesized that CWT analysis will objectively identify FOG better than the traditional method using power spectral analysis by incorporating appropriate sampling periods and update time with optimal sensor number/placement locations.

## 2. Materials and Methods

The present work consists of novel analyses performed on the complete dataset obtained from previously published data [16]. Bachlin and colleagues recruited 10 PD patients (three females; age: 66.5 ± 4.8 years; disease duration: 13.7 ± 9.67 years; H & Y in ON: 2.6 ± 0.65) with the history of FOG and could walk during the “off-medication” stage without additional assistance. All participants except for two individuals (who had frequent FOG experience during the “on-medication” state) performed the tasks in the “off” stage of medication.

Different walking conditions were provided to the participants (walking back and forth in a straight line, random walk with several stops and 360° turns, and walking simulating activities of daily living). All trials were recorded on a video camera. Two physiotherapist analyzed the videos of the patients to detect four different activities: walking, standing, turning, and freezing. The term no-freeze included the activities of walking, standing, and turning. For each FOG episode, they specified the start and end times of FOG. Additionally, three 3-D accelerometers were used to collect the accelerations of shank (just above the ankle), thigh (just above the knee), and lower back. The accelerometers were sampled at 64 Hz sampling rate. In this dataset, there were totally 8 h and 20 min of data with 237 FOG events which were identified by the physiotherapists.

Previous studies [15,16] used Fourier transform to elicit information from the frequency domain of acceleration data in order to detect the FOG episodes. In this study, we applied CWT on acceleration data to extract further features. CWT method provides the information not only in frequency domain but also in temporal domain that may help in defining a better FOG index using an appropriate window size and update rate.

The CWT constitutes a set of scaled and shifted wavelets in the frequency and time domain, respectively [17,18]. Each wavelet is generated by mother wavelet and has a finite length with zero average. Each wavelet could be constructed by stretching or compressing (s, scale) the mother wavelet and transferring it in temporal domain (τ, translational time). The CWT of the acceleration signal (a(t)) was defined as the integral of multiplication of acceleration signal and wavelets over the duration of window:

(1) $$C\left(s,\tau \right)=\int_{0}^{\text{window}}a\left(t\right)\Psi^{*}\left(\frac{t-\tau }{s}\right)\text{dt}$$

where Ψ is a mother wavelet function. $\Psi^{*}\left(\frac{t-\tau }{s}\right)$ is the conjugate of mother wavelet which is shifted in temporal domain and scaled. The CWT of acceleration signal (C(s, τ), CWT coefficients) is the function of both scale and translational time. Since the scale is inversely proportional to the frequency, the corresponding pseudo-frequency for a specific scale could be computed by:

(2) $$F_{s}=\frac{F_{c}}{s.\Delta }$$

where *F_c_* is the center frequency of mother wavelet. Δ is the sampling time in data collection from hardware specification and *F_s_* is the pseudo-frequency which corresponds to a specific scale (*i.e.*, s). Throughout the manuscript, we will use the term “frequency” to refer to pseudo-frequency.

During FOG there were frequency components in 3–6 Hz [12]. Accordingly, we defined two scale ranges to capture the spectral components separately. The two scale ranges which correspond to frequency ranges of 0.5–3 Hz and 3–8 Hz were considered as locomotor and freeze scales, respectively. We computed the locomotor (LC(τ) in Equation (3)) and freeze (FC(τ) in Equation (4)) components at each translational time as the summation of CWT coefficient values of correspondent scale ranges. Therefore, we introduced the percentage ratio (R(τ) in Equation (5)) of locomotor component to the sum of locomotor and freeze components. The FOG index was defined as the average of R(τ) over the sampled window.

(3) $$\mathrm{Locomotor}\mathrm{component}:LC\left(\tau \right)\;=\;\sum_{i=0}^{5}\;C\left(s\;=\;\frac{F_{c}}{0.5\left(1+i\right)\times \Delta }\;,\;\tau \right)$$

(4) $$\mathrm{Freeze}\mathrm{component}:\text{FC}\left(\tau \right)=\sum_{i=5}^{15}C\left(s=\frac{F_{c}}{0.5\left(1+i\right)\times \Delta }\;,\tau \right)$$

(5) $$\mathrm{Ratio}:R\left(\tau \right)=\frac{\text{LC}\left(\tau \right)}{\text{LC}\left(\tau \right)+\mathrm{FC}\left(\tau \right)}\;\times \;100$$

As we mentioned above, the two frequency ranges of 0.5–3 Hz and 3–8 Hz were considered for locomotor and freeze components, respectively. We tried different step sizes (e.g., 0.1, 0.25, 0.5, 1, 1.5 Hz) in frequency ranges for calculating the locomotor and freeze components. For any step size above 0.5 Hz, the frequency resolution was not enough to lead to different values for locomotor and freeze components. So, these step sizes were not good at discriminating these two components. However, the discrimination results were obtained by choosing any step sizes equal or below 0.5 Hz. Therefore, we chose 0.5 Hz as the proper step size. Consequently, we needed to calculate CWT coefficients for 6 and 11 frequencies (or scales) in order to compute the locomotor and freezing components, respectively. Note that 0.5 in the denominators of Equations (3) and (4) is the step size in frequency. As in Equation (2), we could calculate the correspondent scale of each frequency. For example, the term of 0.5(i + 1) for the values of i = 0, 1, 2, …, 5 generates the frequencies of 0.5, 1, 1.5, …, 3 Hz in the denominator to select the correspondent scales of these 6 frequencies for locomotor component in Equation (3).

In the current study several sample window sizes (1 s, 2 s, 3 s, and 4 s) have been applied to assess the effects of sampling duration on the FOG detection with CWT method. The same sample window size was always used for the locomotor and freezing components in order to calculate the ratio. The smallest sample window size in previous studies [16] were 4 s. We also want to assess smaller window sizes in order to not miss the short FOG episodes.

The optimal decision threshold for discriminating FOG from the other activities was selected according to the accuracy of diagnostic tests using an interactive dot diagram in MedCalc software (MedCalc statistical software, version 13). In the graph, we defined two groups “FOG” and “No FOG” (the other activities) which were plotted on two vertical axes by dots. A horizontal line in the graph indicated a threshold which illustrates the best separation between the two groups (via obtaining minimal false negative and false positive results).

The signals of three axes in each accelerometer were considered as independent variables. These signals were low-pass filtered using a fourth order, zero lag, Butterworth filter at a cut off frequency of 10 Hz. The FOG index for each axis was calculated. We assumed the clinical assessment from [16] as the ground-truth and assessed the performance of FOG index via sensitivity and specificity criteria. The sensitivity was the probability that the index detected FOG when FOG was present. Specificity indicated the probability that the index detected normal movement when there was not a FOG.

The type of the mother wavelet (Ψ(t)) could also influence the CWT coefficients. We chose db4 (Daubechies wavelets of 4th order) as the mother wavelet in the current study [19,20]. We applied CWT on 2 subjects with other wavelet families and orders (e.g., db10, Morlet, Haar, Mexican Hat, and Gaussian) to find the appropriate wavelet. Our results showed that db4 could discriminate locomotor and freezing components better and gave a larger values for the sensitivity and the specificity.

## 3. Results

**Figure 1:**
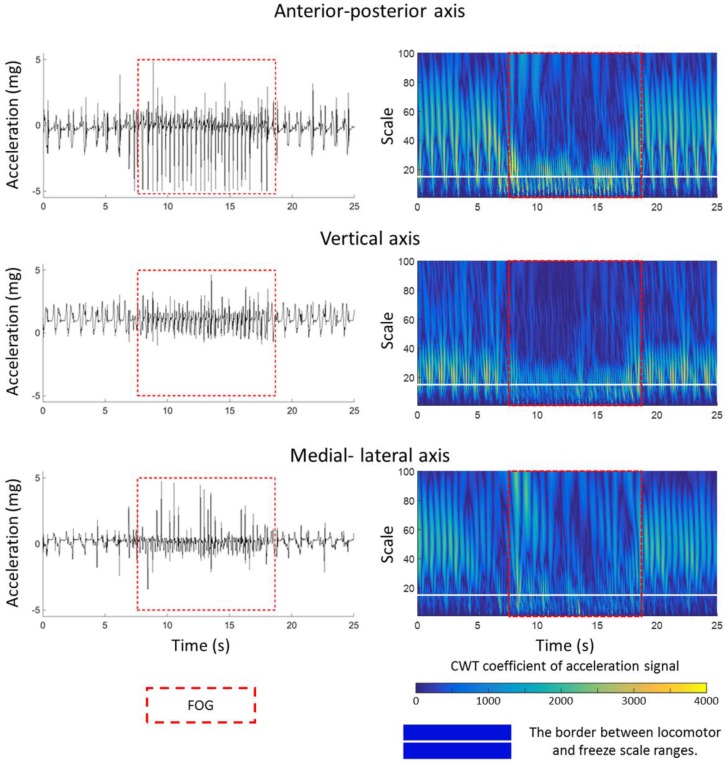
Shank acceleration signal and corresponding continuous wavelet transform of 25 s signal extracted from subject 2. The red dash rectangles denoted the true freezing of gait (FOG) episode period which physiotherapists identified by watching the video of a patient during the trials. At each continuous wavelet transform (CWT) plot, the white horizontal dash line indicated scale 15.2 which corresponded to 3 Hz and defined the border between locomotor and freeze scale ranges. The upper and lower sides of the white line indicated the locomotor and freeze scale ranges which corresponded to frequency ranges of 0.5–3 Hz and 3–8 Hz, respectively. The frequencies of 0.5, 3, and 8 correspond to the scales of 91.4, 15.2, and 5.7, respectively. Milli-gravitational acceleration is denoted by mg (980.665 mm/s^2^).

The CWT coefficient values are plotted in Figure 1. The profile of accelerations changed during FOG episode (left column, Figure 1) and CWT of accelerations (right column, Figure 1) magnify these changes in order to capture them computationally better. The inside of the red dash rectangle box specifies the FOG episode. The white horizontal line discriminates the two scale ranges of locomotor and freezing activities. In other words, below and above the white line corresponds directly to the frequency ranges of 3–8 Hz and 0.5–3 Hz, respectively. The CWT coefficients in Figure 1 shows relatively larger values (lighter blue values appeared in the below of white line) in the scales corresponding to the pseudo-frequency 3–8 Hz during the FOG episode (in the red dash rectangle box) in comparison to other activities (out of the red dash rectangle box) in all three axes of shank accelerometer. In contrast, the CWT coefficients had larger values in the pseudo-frequency 0.5–3 Hz (lighter blue values appeared in the above of white line) during the normal movement.

**Figure 2:**
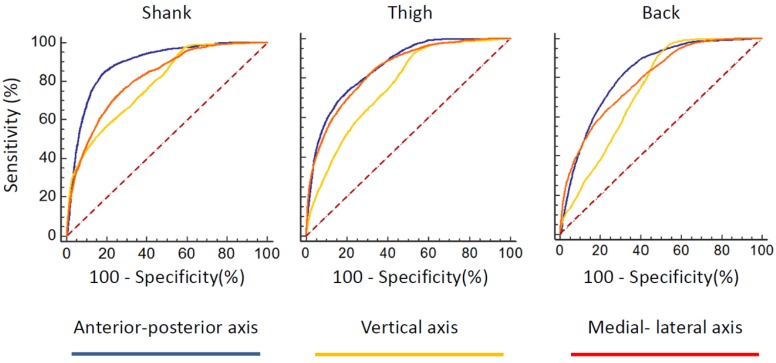
Receiver operating characteristic (ROC) curves for all sensors and all the three axes.

The receiver operating characteristic (ROC) curves of FOG index for all sensors and all of the three axes were provided in Figure 2. According to the area under the ROC curve, anterior–posterior direction could discriminate FOG better than the other two axes in any sensor placement locations (Table 1). As such, the anterior–posterior axis of acceleration signal maybe a good candidate for detecting the FOG.

**Figure 3:**
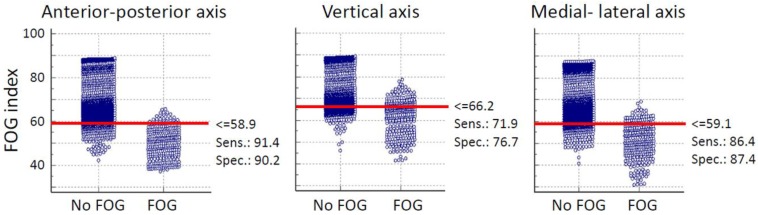
Interactive dot diagram of the FOG index for the shank senor of subject 2 for three different axes.

The threshold which discriminated FOG from the other activates was defined from the average data of all subjects based on the results of interactive dot diagram. The interactive dot diagram of FOG index for shank senor of subject 2 is illustrated in Figure 3. The horizontal line in each graph shows the best threshold which could discriminate FOG from other activities. In Figure 3, each circle shows the value of the proposed FOG index which are classified in two groups based on our ground truth clinical assessments. So, all circles in the “FOG” group indicate true FOG events and those in the “No FOG” group are true no FOG events (other movements). The overlap between the two sections indicate the false negative (circles located above threshold line in “FOG” group) and false positive (circles located below threshold line in “No FOG” group) of the proposed index.

Furthermore, in terms of the real-time detection of the FOG episodes, the assignment of the window size and update time may be critical. Utilizing the results [16], we used the window length of 4 s with the update time of 0.5 s and applied CWT to all three sensors locations in order to find the best sensor position for detecting the FOG event. The averages of sensitivity and specificity across all subjects are presented in Table 2. The results indicate that the shank was the best placement location to detect FOG episodes rather than lower back or thigh positions.

**Figure 4:**
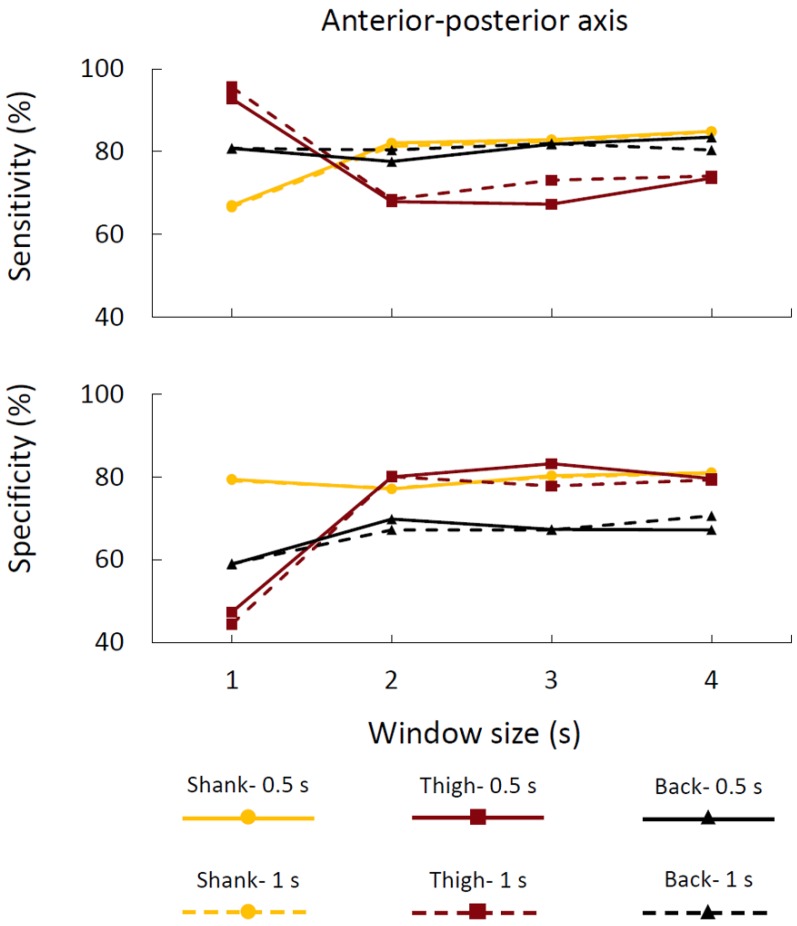
The averages of sensitivity and specificity across all subjects as a function of window size, update time, and sensor placement.

In order to verify the appropriate window size and update times, we applied the FOG detection method on various window sizes that were smaller than 4 s (3, 2 or 1 s) on the anterior–posterior acceleration of the different sensor placements. We also employed larger update time (1 s) to examine the case in which the access to the processor could be limited and fast calculation was not available which would require the larger update time. The averages of sensitivity and specificity of various window sizes, update times, and sensor placements were presented in Figure 4.

The results indicated that the acceptable sensitivity and specificity on the anterior–posterior shank acceleration sensor was achievable even for shorter window sizes (*i.e.*, 2 s; Figure 4) by employing the proposed FOG index. Importantly, the proposed index showed strong robustness to the update time variability. In other words, the sensitivity and specificity were robust to the changes of update time from 0.5 s to 1 s.

One of the design aspects in online detection is the number of false positive rates that may influence user compliance. Since we had different sample window sizes and update times, we provide average false positive percentages of the test results per minute for FOG index in Table 3.

## 4. Discussion

We applied the CWT on wireless accelerometer signals to acquire real-time FOG detection. The novel FOG index was created to capture the time and frequency components of acceleration signal. To the best of our knowledge, this paper is the first attempt to utilize CWT for real-time detection of FOG events. Additionally, we evaluated the optimal sensor placement locations for identifying FOG. The efficiency of real-time detection of FOG was further investigated by assessing window size and update time. These two variables can be highly important when real time feedback (e.g., auditory, vibratory stimulations) is applied in order to overcome FOG [16,21,22].

The current findings from applying CWT to linear acceleration signals were compared with previous studies [12,15] and it was found that there was an increase in amplitudes in the frequencies from 3–8 Hz in comparison with normal movements during FOG.

Very small shuffling steps with minimum forward movement and leg trembling in place (some leg trembling but no effective forward motion) were the most common manifestations of FOG [23]. The previous studies concentrated only on leg trembling in place [6,23] and used the frequency information of acceleration signal in the vertical axis which was the axis where the trembling occurred. The current study showed that we could also distinguish FOG by using the capability of the CWT to capture information from both time and frequency domain (Figure 1). Interestingly, the novel FOG index could detect FOG in anterior–posterior axis better than the other two axes. Since the shuffling steps had minimum forward movement and the axis of minimum forward movement corresponded to anterior–posterior axis, the proposed index could discriminate FOG in anterior–posterior better.

The proposed FOG index detected FOG with 84.9% sensitivity and 81.01% specificity for sensor placed at shank with a window size of 4 s and update time of 0.5 s. Using our method, the sensitivity was much higher than the previously reported result of 73.1%, applying FFT to the same dataset, but the specificity was similar (81.6%) [16].

In terms of the location of the sensor placements, shank and lower back positions were preferred as these locations did not interfere with normal walking and produced best results. However, the lower back position could not represent trembling in place effectively during FOG events. As such, detection of FOG events using the shank sensor was better than the lower back sensor location (Figure 4). These results were in agreement with the other studies which evaluated sensor placement at the lower back for FOG detections [7,24]. In conclusion, a single sensor at the shank location may be the best sensor placement location for identifying FOG events, as this sensor location will not interfere with gait and allows easy installation of the sensors.

In the current study, 48.1% of FOG events lasted less than 5 s. Thus, it was necessary to detect the FOG events with a shorter window size. Additionally, the efficiency of real-time detection could be improved with lowering sampling window and increasing update time.

None of the previous studies have reported false positive per minute of their method. This value should be considered during early design phase since false positive signals will limit the usability of the device (*i.e.*, “cry-wolf” effect for user compliance—users may turn the system off if too many false alarms are engaged). Based on our results from Table 3, false positive per minute values might be high from the design aspect, but it should be considered that these results were obtained from global threshold (all subjects have same threshold). By determining the threshold for each subject (*i.e.*, calibrate index for each individual), the false positive rate will notably be decreased. Since we didn’t have access to other activity conditions (walking, standing, and turning), we were limited to improve our proposed index to identify FOG events from all other conditions.

The current framework (window size of 2 s and update time of 1 s) achieved the levels of 82.1% and 77.1% for sensitivity and specificity, respectively. This framework had several special characteristics: First, the smaller window size, such as 2 s, allowed detection of short-duration FOG episodes better than a larger window size, since the larger window size can average out shorter FOG episodes. Second, the smaller window size could also decrease the processing time and send the signal to the third party faster (e.g., an assistive device) to help patients overcome the FOG episodes. Third, the robustness due to the update time variability did not attenuate the levels of detection. These characteristics are promising for real time application as these settings will decrease the calculation time and increase the efficiency.

## 5. Conclusions

The current work indicates that real-time detection of FOG using CWT of acceleration data is attainable with a small window length of 2 s and the larger update time of 1 s using the single shank sensor. Since a single shank sensor did not interfere with walking and was placed easily, it facilitated gait analysis as well as real-time FOG detection. Although the requirements for FOG detection were well resolved, future studies should implement and examine the utility of this method in real-time detection of FOG and associated feedback stimulations. In other words, future studies should implement this method in a real time fashion to validate the results.

One of the limitations of this work was that the data-sets were demarcated as only freezing and non-freezing conditions, limiting the details about non-freezing conditions such as walking, standing, and turning. Given this information, detection of FOG events could be better ascertained by characterizing and distinguishing other non-freezing features/activities prior to applying CWT.

## Figures and Tables

**Table 1:** Area under the ROC curve across all the subjects for the different sensor positions and axes.

Sensor Position	Anterior–Posterior	Vertical	Medial-Lateral
Shank	0.890	0.786	0.815
Thigh	0.857	0.759	0.842
Lower back	0.821	0.738	0.793

**Table 2:** Sensitivity, specificity, and area under ROC curve across all the subjects for the different sensor positions with the time window size 4 s and the update time 0.5 s.

Sensor Position	Sensitivity (%)	Specificity (%)	Area under ROC Curve
Shank	84.9	81.0	0.890
Thigh	73.6	79.6	0.856
Lower back	83.5	67.2	0.821

**Table 3:** Average false positive percentage of the test results per minute for FOG index, across all the subjects for the anterior–posterior axis of shank senor.

Sample Window Size (s)	Update Time 0.5 s (%)	Update Time 1 s (%)
4	16.42	16.35
3	16.52	17.05
2	15.49	18.11
1	15.40	17.78

## Abbreviations

The following abbreviations are used in this manuscript:

PD	Parkinson’s disease
FOG	Freezing of gait
CWT	Continuous wavelet transform
ROC	Receiver operating characteristic
NINDS	National Institute of Neurological Disorders and Stroke
IMU	Inertial monitoring units
